# Prevention is better than cure: why early interventions for insomnia and chronic pain during adolescence should be a priority

**DOI:** 10.3389/fpsyg.2023.1206977

**Published:** 2023-06-08

**Authors:** Tor Arnison

**Affiliations:** Clinical Epidemiology and Biostatistics, Faculty of Medicine and Health, School of Medical Sciences, Örebro University, Örebro, Sweden

**Keywords:** prevention, adolescents, insomnia, chronic pain, early interventions, psychological treatment

## Introduction

The onset of both chronic pain (King et al., 2011) and insomnia (Donskoy and Loghmanee, 2018) is high during adolescence. In fact, the prevalence of new-onset chronic pain is higher in adolescents than in any other age group (King et al., 2011). This phenomenon is believed to be caused by a range of biological (e.g., hormonal and neurophysiological changes), psychological (e.g., changes in cognitive and emotional processes related to pain), and social factors (e.g., changes in social identity and peer and family dynamics) that are unique or particularly salient during adolescence (Christensen et al., 2019): Adolescence is a developmental period characterized by the transition from being a child to being an adult (Santrock, 2014). This includes biological changes through puberty, which also leads to stormy and strongly felt emotions. These are particularly difficult for adolescence to handle since their ability to regulate emotions are yet to be fully developed (Riediger and Klipker, 2015). Adolescents also stand before a number of large decisions and processes—they are to develop their own identity in the world, lay the foundation for their professional career, and become independent persons by distancing themselves from their families while becoming more dependent on friends and various social groups (Santrock, 2014). All these factors naturally constitute sources of major stress for the adolescent. In addition, sleep architecture undergoes changes during adolescence, for example demonstrated by a significant shift in sleep phase toward later sleep-wake patterns (Hagenauer et al., 2009). In combination with early school times, this causes chronic sleep deficiencies in many adolescents since they, due to their delayed circadian rhythm, fail to fall asleep early enough to get sufficient sleep (Zerbini and Merrow, 2017).

## Insomnia and chronic pain in adolescents

The prevalence of both chronic pain and insomnia is high in adolescents. As high as 40 percent of adolescents suffer from either chronic pain (Miro et al., 2022) or insomnia (de Zambotti et al., 2018). In addition, chronic pain and insomnia co-occur to a high degree in adolescents (Arnison et al., 2022a): more than 50% of adolescents with chronic pain also suffer from insomnia (Palermo et al., 2011), and as comorbid conditions, they appear to cause more severe symptom profiles of illness compared to each of the conditions on their own (Palermo et al., 2012). A worrisome pattern is that the prevalence and severity of chronic pain seem to have increased considerably across the last 20 years (Miro et al., 2022; Roy et al., 2022). Chronic pain or insomnia in adolescents is furthermore at high risk of persisting into adulthood (Walker et al., 2010; Kashikar-Zuck et al., 2019; Hysing et al., 2020), where they both are debilitating, leading causes of disability and among the major health concerns in the world (Wallander et al., 2007; Donskoy and Loghmanee, 2018; James et al., 2018; Mills et al., 2019). The adolescent brain undergoes major changes in structure and functioning, which makes them particularly sensitive to environmental and behavioral influences (Spear, 2013), and neurological brain circuits gradually become less plastic and more permanent across adolescence and moving into adulthood (Palermo, 2020). This pattern may help explain why adolescents are particularly vulnerable to developing both chronic pain and insomnia, as well as why they to such a high degree carries over into adulthood where they are notoriously difficult to treat (especially chronic pain).

Importantly, the relationship between chronic pain and insomnia appears to be bidirectional in adolescents (Lewandowski et al., 2010; Arnison et al., 2022b; Liu et al., 2022; Albinni et al., 2023), such that the conditions may reciprocally influence each other in a downward spiral of increasing suffering and disability. An interesting and important finding, although perhaps not intuitive, is that sleep problems seem to inert a significantly stronger effect on chronic pain than the other way around (Finan et al., 2013; Arnison et al., 2022b).

## Proposed explanatory mechanisms of the insomnia-pain relationship in adolescents

So, what may explain the relationship between insomnia and chronic pain? It is generally believed to be a biopsychosocial relationship (Whibley et al., 2019), and several theories have been proposed: the majority of these have been psychophysiological, attempting to explain the relationship through neurophysiological systems that affect insomnia, pain, as well as psychological factors (Herrero Babiloni et al., 2020). Negative mood or affect have consistently been shown to mediate the sleep-pain relationship (Pavlova et al., 2017; Whibley et al., 2019), and, for example, the opioidergic, dopaminergic and monoaminergic systems are all reciprocally associated with sleep, pain and negative mood (Haack et al., 2020; Herrero Babiloni et al., 2020). Other examples are melatonin and adenosine (Haack et al., 2020), systemic inflammation (Herrero Babiloni et al., 2020), and hypothalamus-pituitary-adrenal (HPA) axis dysfunction (Herrero Babiloni et al., 2020; Albinni et al., 2023). Maladaptive emotion regulation may also explain the relationship between insomnia and pain (Albinni et al., 2023), since sleep disturbance has a negative effect on emotion regulation (Palmer and Alfano, 2017), which, in turn, has a negative effect on pain (Koechlin et al., 2018). Although sparsely researched, social factors may also explain the relationship. For example, both insomnia and pain may influence social activity and social isolation in a negative spiral (Andreucci et al., 2021), and thereby not only enhance the negative effects of social isolation, but also hamper the protective effects of social support.

## Practical implications

As mentioned, onset of both insomnia and chronic pain increases considerably during adolescence. In addition, they persist into adulthood to a high degree, where they (particularly chronic pain) are difficult to treat. Although this is a very unfortunate pattern, one may find a silver lining, or rather an opportunity, in the prospect of prevention: adolescence may be considered an essential developmental stage to develop and implement early interventions and preventive strategies to prevent insomnia and chronic pain from developing and becoming chronic.

Since insomnia is a stronger predictor of pain than vice versa, it may be most constructive to focus on insomnia in such early interventions. Especially considering that effective treatments for insomnia already exists, such as Cognitive Behavior Therapy for Insomnia (CBT-I; Selvanathan et al., 2021). This also seemed to be the conclusion several reviews on the topic have come to (Smith and Haythornthwaite, 2004; Finan et al., 2013, 2014; Tang et al., 2015), after reviewing treatment studies on comorbid insomnia and pain. Today, there is robust evidence showing that insomnia-focused psychotherapeutic treatments have strong effects on insomnia symptoms, and small to moderate effects on chronic pain (Selvanathan et al., 2021). As an attempt to improve the efficacy of treatments for comorbid insomnia and chronic pain, hybrid treatments, which include elements from treatments for both insomnia and chronic pain, have been proposed as a prospect. Such hybrid interventions have shown promising, albeit inconclusive, results in adults (Tang, 2018). Regarding adolescents, there is rather limited evidence for interventions targeting comorbid insomnia and chronic pain, although few published studies have shown promising results (Palermo et al., 2017; Law et al., 2018). Hybrid treatments are yet to be developed and implemented in adolescents.

Even though it is well-established that an increase in insomnia can predict an increase in chronic pain, and vice versa, the evidence is less clear regarding if a decrease in insomnia may predict a decrease in chronic pain (Afolalu et al., 2018; Arnison et al., 2022a). Although more research is needed on this topic, one hypothesis is that insomnia and (especially) chronic pain, once the conditions turn chronic, become more independent conditions where their reciprocal relationship is weakened. If this hypothesis were to be true, it would only further emphasize the importance of early interventions during adolescence before the conditions have become chronic.

## Discussion

Due to the critical developmental stage adolescence constitutes for the co-development of insomnia and pain, it is imperative to conduct more research on how insomnia treatments in adolescents may prevent chronic pain from developing and carrying over into adulthood. This would necessitate a range of studies, such as randomized-controlled trials, as well as longitudinal studies spanning multiple years, and preventive school-based programs, assessing what effect interventions in adolescence may carry over into adulthood.

An approach that may be particularly promising in adolescents are school-based prevention programs. This is especially true for programs addressing insomnia and sleep deficits in adolescents (Cassoff et al., 2013), which may indirectly also prevent chronic pain. However, emerging evidence suggests that also school-based pain-prevention programs may be effective (Nilsson et al., 2019). Hybrid school-based interventions, combining elements from sleep- and pain-focused prevention programs, might be a viable method to prevent insomnia and chronic pain from developing in adolescents.

Regarding early interventions for preventing insomnia and pain from establishing and becoming chronic in adolescents, CBT-I is a promising prospect. However, since unique changes in sleep and circadian factors occur during adolescence (Palmer, 2020), it may not suffice to simply transfer CBT-I for adults to adolescents. For example, adolescents show a pronounced delay in sleep phase which, in combination with early school times, causes many adolescents to be chronically sleep deprived (Carskadon, 2011). A novel treatment protocol, the Transdiagnostic Sleep and Circadian Intervention (TranS-C), addresses this issue. It is a hybrid treatment protocol that also includes a focus on sleep phase (Dong et al., 2019). Importantly, the protocol is aimed at targeting comorbidities with insomnia as treatment outcomes (Harvey et al., 2016), which includes chronic pain. The protocol has, to date, limited but promising effects on adolescents (Dong et al., 2019). Future studies should examine the feasibility of implementing TranS-C, or similar treatments, for adolescents with insomnia at risk of developing chronic pain.

Furthermore, although hybrid interventions, combining elements from both CBT-I and Cognitive Behavior therapy for Chronic pain (CBT-P), are promising, there might be further untapped potential in the hybrid approach. Given that negative mood and maladaptive emotion regulation strategies, such as rumination increase during adolescence (Nolen-Hoeksema, 2000), and they are associated with both insomnia and pain (Edwards et al., 2011; Palmer et al., 2018; Whibley et al., 2019), a promising venue is to extend future hybrid treatments for comorbid chronic pain and insomnia in adolescents. Such hybrid treatments could, apart from treatment modules from CBT-I and CBT-P, also incorporate treatment elements targeted more specifically at negative mood and emotion regulation. For example, the Unified Protocol is a modular and transdiagnostic treatment protocol focused at improving (maladaptive) emotion regulation abilities (Barlow et al., 2017). It has proven effective as treatment for a range of psychiatric disorders (Barlow et al., 2020) and has been adapted for adolescents (Ehrenreich-May et al., 2017). Incorporating components from a transdiagnostic treatment protocol such as the Unified Protocol into hybrid treatments for insomnia and chronic pain may, as a secondary effect, also treat comorbid psychiatric symptoms such as depression and anxiety. In fact, both sleep problems (Owens et al., 2014; Asarnow and Mirchandaney, 2021) and chronic pain (Palermo, 2020) predict mental illness in adolescents. Targeting insomnia and chronic pain in adolescents may therefore also prevent mental illness in a broader sense.

An important factor, which to date have received limited attention (Albinni et al., 2023), is sex or gender differences in the insomnia-pain relationship. Both insomnia and pain are considerably more prevalent in females, and these differences first arise during adolescence (de Zambotti et al., 2018; Christensen et al., 2019). Emerging evidence demonstrate that the effect of insomnia on pain may differ depending on sex (Zhang et al., 2012; Smith et al., 2019). Because of this, it is also imperative to consider interventions that are uniquely developed for adolescent girls and boys, separately. Not doing so may confound and impair the potential effects of interventions for insomnia in adolescents.

Although the effects of sleep problems on chronic pain likely is a constant issue in adolescents, it may have become particularly salient in the wake of the COVID-19 pandemic. Indeed, concerns have been raised regarding the risk of chronic pain prevalence increasing because of the pandemic (Clauw et al., 2020). This may partly occur due to COVID-19 infection itself, since new-onset chronic pain is one of the main manifestations of long COVID (Shanthanna et al., 2022). It may partly occur due to secondary consequences of the pandemic: social distancing, for example, has led to increased social isolation and inactivity, and to adolescents spending more time in bed, with lower quality of sleep, as compared to before the pandemic (Richter et al., 2023). Symptoms of mental ill-health, such as depression and anxiety, has increased in the adolescent population (Gotlib et al., 2022), including in youths who suffer from chronic pain (Birnie et al., 2022). Therefore, preventing insomnia and chronic pain in adolescents may be particularly important in the aftermath of the pandemic.

Taken together, adolescence is a boiling pot of factors making the co-development of insomnia and chronic pain particularly salient. At the same time, adolescence constitutes a promising developmental period to implement preventions or early interventions for comorbid insomnia and chronic pain, which may prevent the conditions from becoming chronic and difficult to treat in adulthood. I therefore call for further research on prevention and treatment for comorbid chronic pain and insomnia in adolescents.

## Author contributions

The author confirms being the sole contributor of this work and has approved it for publication.
